# The Practice Market.—VIII
*Previous articles of this series appeared in The Hospital of March 18, March 25, April 1, April 29, June 17, June 24, and July 15.


**Published:** 1911-08-26

**Authors:** 


### SPECIAL ARTICLE.

### THE PRACTICE MARKET.—VIII.*

### THE INVESTIGATION OF A MEDICAL PRACTICE.

We said in our last article that the prospective purchaser, if he is satisfied from the scanty preliminary particulars sent by the agent that a given opening is worth looking into, should immediately apply for further information, preferably by a personal interview with the head of the agency. If this, so far as it goes, is satisfactory, he should arrange for an early visit to the vendor. An hour or two's talk with the principal in his own house, following an intelligent interview with the agent, should enable the inquirer to decide whether or not it is useless to go any deeper into the matter. If after the preliminary visit he is not inclined to negotiate further, he should at once inform the vendor and the agent of the fact. If, on the other hand, he is favourably impressed with the opening, he should lose no time in arranging to see something of the practice at work and in making such inquiries locally and otherwise as may be agreed to by the principal. The better the opening the more desirous will the vendor be to facilitate the fullest investigation by a *bonâ-fide* inquirer into its merits and prospects.

#### Lines of Investigation.

Among the many points which the purchaser should inquire into when examining a practice we will mention those which seem to us to be the most important. He should ask why the vendor is giving up practice or taking in a partner; how long he has been in the practice; and what is the nature and extent of the local opposition. He should inquire closely into the different elements of the practice, and the proportions of the various classes of patients; also into the radius of the practice, the means of locomotion, and the frequency of midwifery and night work generally. If the rough preliminary financial statement of the practice shows wide recent fluctuations in the receipts, an explanation of these should be requested. The far-reaching provisions of the National Insurance Bill make it imperative that the prospective buyer should demand a full account of any club or dispensary work included in the practice, as well as an estimate of the number of persons on the vendor's private books who will be compelled to insure for medical benefits under the proposed scheme.

#### The Insurance Bill and Medical Transfer.

The revolt of medical practitioners and the anxiety which is felt throughout the profession have already interfered very considerably with the business of medical transfer, especially in industrial districts and in practices which depend to any appreciable extent on Friendly Society appointments. At this crisis in the history of the medical profession it is vitally important to the man who contemplates investing his capital in general practice that he should realise the possibilities of the Bill and conduct his inquiries and his negotiations accordingly. If the Bill is passed as it stands there will be a great readjustment of the conditions of general practice, and a large number of practices can scarcely fail to be affected adversely. Without wishing to sound an unnecessary note of alarm, we feel convinced that the near future is likely to be an extremely critical time, alike for vendors, for purchasers, and for middlemen in the Practice Market. The purchaser especially must be very careful.

Further matters which should be inquired into include the proportion of surgical and special work in the practice, and the opportunities for such practice which will be available for a newcomer: these will raise the question of the presence of a cottage hospital. Information must be sought about the arrangements in the practice for dispensing—whether this is done by the practitioners themselves, or by a dispenser, or by arrangement with a chemist, or simply by prescriptions; and here again the Insurance Bill may profoundly alter the present conditions of general practice.

#### Business Points.

The vendor's method of business should be inquired into—how he keeps his books, how often he sends in his accounts, and who does his bookkeeping. A business-like practitioner can usually produce at a moment's notice an annual statement of accounts and balance-sheet for all the years he has been in practice, showing the proportions of expenses and receipts under various inclusive headings. The question of house accommodation for the incomer must receive early attention: the vendor usually has a definite proposal for the optional or obligatory taking over of a given residence by the successor or junior partner, as the case may be. The purchaser must ask about the situation of the house with regard to the area of the practice, and about its rent and rates and the proposed length of tenancy, and of course about its accommodation, age, and state of repair.

The general terms of transfer must of course be settled, with special reference to the price, to the status of the incomer, and to the length of introduction in the case of succession. It cannot too forcibly be stated that no practice or share should ever be bought until the books of the practice have been examined by a professional accountant, and preferably by one who is familiar with this kind of business. As soon as the principals have come to some sort of preliminary terms, and before any draft agreement is drawn up by the solicitors, the purchaser should obtain permission from the vendor for a full expert investigation of the books for some years past. No *bonâ-fide* vendor can possibly object to this, but the purchaser should not go to the expense of sending down an accountant until there is a strong probability of a settlement being arrived at.

#### A Last Caution.

Medical men vary greatly in their business habits: many are poor men of affairs, haphazard,

* Previous articles of this series appeared in The Hospital of March 18, March 25, April 1, April 29, June 17, June 24, and July 15.

unmethodical, and easy-going. There are others the very reverse of this—keen, shrewd business men, always "on the make," determined to come off best in every bargain, and quite ready to overreach anyone who is fool enough to neglect his own interests. When a doctor has the commercial instinct he is usually as hard and as sharp a customer as can be found anywhere, and the keenest business heads in the Practice Market often go with a deceptive urbanity and readiness of tongue. Let the buyer beware. However obvious his need for warning and protection, the agent's lips are sealed, and he must trust to legitimate local and professional inquiries, and to his own accountant and solicitor, for guidance in this all-important matter. No medical man is competent to deal unaided in the Practice Market beyond a certain point, whether as buyer or as seller. If this series of articles has made that one fact clear, it has not been written in vain.

